# 17.2 EFFICACY OF CANNABIDIOL IN THE TREATMENT OF EARLY PSYCHOSIS.

**DOI:** 10.1093/schbul/sby014.066

**Published:** 2018-04-01

**Authors:** Mohini Ranganathan, Deepak D’Souza, Jose Cortes-Briones, Patrick Skosnik

**Affiliations:** 1 Yale University School of Medicine; 2 Yale University School of Medicine, VA Connecticut Healthcare System

## Abstract

**Background:**

Cannabidiol is a component of herbal cannabis, studied for a number of potential pharmaceutical indications, more recently, its potential anti-psychotic effects with an extremely favorable side effect profile. Cannabidiol content of cannabis may also attenuate the psychotic and cognitive effects associated with cannabis use. Early psychosis is associated with alterations in the endocannabinoid system and is marked by limited engagement in treatment, reluctance to use traditional antipsychotics, sensitivity to medication side effects and heavy cannabis use. Cannabidiol may thus represent a more acceptable and tolerable antipsychotic medication in this phase of illness with a novel mechanism of action.

**Methods:**

Data will be presented from an ongoing double blind, placebo controlled, within subject, crossover study examining the effects of Cannabidiol (800mg/day) versus placebo in individuals within the first 7 years of their psychotic illness. Subjects participate in two treatment periods, each four weeks long separated by at least 2 weeks of washout.

**Results:**

Data will be presented on the effects of Cannabidiol on psychotic symptoms (measured on the Positive and Negative Syndrome Scale), cognitive deficits (MATRICS battery), electrophysiological biomarkers of information processing (Resting EEG and ERPs relevant to psychosis and cannabinoids), metabolic parameters and general functioning.

**Discussion:**

Cannabidiol is a novel drug that has shown potential efficacy in the treatment of psychotic symptoms. Early psychosis is a critical treatment period during which treatment engagement and adherence is critical and duration of untreated psychosis is associated with long term negative consequences. Cannabidiol may thus represent a more acceptable and tolerable medication to target this vulnerable population.

